# The Genetic Landscape of Plasma P-Selectin Glycoprotein Ligand Levels and Bidirectional Mendelian Randomization to Assess Role in Proinflammatory Cytokine Levels

**DOI:** 10.3390/genes17070811

**Published:** 2026-07-16

**Authors:** Christian Bime, Yann C. Klimentidis, Xiaoguang Sun, Chilton H. Floyd, Carrie S. Standage-Beier, Sammani Saad, Nancy G. Casanova, Mathew K. Hufford, Sara M. Camp, Joe G. N. Garcia

**Affiliations:** 1 Division of Pulmonary, Critical Care, and Sleep Medicine, Department of Medicine, College of Medicine, University of Arizona, Phoenix, AZ 85004, USA; ssammani@arizona.edu; 2 Department of Epidemiology and Biostatistics, Mel and Enid Zuckerman College of Public Health, University of Arizona, Tucson, AZ 85724, USA; yann@arizona.edu; 3 Division of Pulmonary, Allergy, Critical Care and Sleep Medicine, Department of Medicine, College of Medicine, University of Arizona, Tucson, AZ 85724, USA; bbxgsun@gmail.com (X.S.); mhufford@arizona.edu (M.K.H.); 4 Department of Nutritional Sciences, University of Arizona, Tucson, AZ 85719, USA; fchilton@arizona.edu (C.H.F.); cstandagebeier@arizona.edu (C.S.S.-B.); 5 Center for Inflammation Science and Systems Medicine, University of Florida Health, Jupiter, FL 33458, USA; n.cassanova@ufl.edu (N.G.C.); jgn.garcia@ufl.edu (J.G.N.G.)

**Keywords:** Mendelian randomization, PSGL1, proinflammatory cytokines

## Abstract

Background: Polymorphonuclear (PMN) leukocyte recruitment to activated pulmonary endothelium is a central mechanism in acute respiratory distress syndrome (ARDS). This process is mediated by selectins and their counter-ligand, P-selectin glycoprotein ligand-1 (PSGL-1), encoded by SELPLG. Genetic variation in SELPLG has been associated with ARDS susceptibility, while disruption of PSGL-1/P-selectin interactions attenuates lung injury in preclinical models. Because inflammatory stimuli increase both SELPLG expression and circulating PSGL-1 levels, PSGL-1 represents a promising biomarker and therapeutic target. We sought to define the genetic determinants of plasma PSGL-1 levels and evaluate their causal relationships with key inflammatory and endothelial biomarkers. Methods: Genome-wide association study (GWAS) summary statistics for plasma PSGL-1 levels were obtained from the UK Biobank Pharma Proteomics Project (*n* = 35,571) and the SCALLOP consortium (*n* = 21,758 across 13 cohorts). Associated variants underwent functional annotation and in silico analyses to identify potential effects on protein structure and gene regulation. Bidirectional Mendelian randomization (MR) was performed using GWAS summary statistics for C-reactive protein (CRP), E-selectin, GlycA, and soluble intercellular adhesion molecule-1 (sICAM-1) to assess potential causal relationships with PSGL-1 levels. Results: Multiple cis- and trans-acting loci were significantly associated with plasma PSGL-1 concentrations. Three coding SELPLG variants (rs201689859, rs74792300, and rs139943851) were predicted to alter PSGL-1 protein structure and were associated with lower circulating PSGL-1 levels. Four promoter variants (rs1420663, rs1833245, rs1420664, and rs8179110) were linked to altered transcriptional activity, including a potential effect of rs1420664 on hypoxia-inducible factor binding. Bidirectional MR demonstrated that genetically predicted CRP, E-selectin, GlycA, and sICAM-1 levels were associated with increased plasma PSGL-1 concentrations. Additional loci implicated pathways related to immune signaling, cell adhesion, and protein stability. Conclusions: Large-scale GWAS and Mendelian randomization analyses identified genetic variants that regulate plasma PSGL-1 levels and demonstrated causal links between inflammatory and endothelial biomarkers and PSGL-1 expression. These findings provide new insights into the genetic regulation of leukocyte trafficking pathways and support a role for PSGL-1 in inflammatory diseases, including ARDS, sepsis, and cardiovascular disorders.

## 1. Introduction

Innate immunity mediates acute inflammation via viral and bacterial infection-induced activation of evolutionary-conserved cascades that lead to increased circulating cytokines/DAMPs, vascular leakage and transendothelial migration (TEM) of polymorphonuclear cells (PMNs) to sites of inflammation [1,2]. Interactions between selectins (E-, L-, P-Selectin) and their counter ligands, including P-selectin glycoprotein ligand 1 (PSGL-1), facilitate PMN trafficking, platelet aggregation, intravascular thrombosis, microangiopathy, and increased vascular permeability during inflammation [3,4,5]. We have previously shown that the selectin P ligand gene (*SELPLG*), which encodes PSGL1, exhibits single nucleotide polymorphisms (SNPs) that confer increased susceptibility to acute respiratory distress syndrome (ARDS), the most severe inflammatory lung injury scenario [6]. Plasma PSGL1 levels were significantly elevated in sepsis, ARDS, and COVID-19 pneumonia compared to controls [7].

In preclinical murine models, *SELPLG* lung tissue expression was significantly increased by key ARDS stimuli LPS, mechanical ventilation) with inflammatory lung injury significantly attenuated by either *SELPLG* knock down or by PSGL1 inhibition [6]. ARDS stimuli significantly increase *SELPLG* promoter activity that is regulated by reactive oxygen species (ROS)-related transcription factors such as HIF1α/2α and NRF2 and by DNA methylation/demethylation [8]. As haplotypes of Selectin P (*SELP*) and *SELPLG* are associated with increased risk of incident coronary artery disease [9,10] and stroke [10], plasma PSGL-1 levels may be viewed as a biomarker for inflammation.

The present study, leveraging two large population-based genome-wide association (GWAS) studies with availability of plasma protein measurements, attempts to address dual objectives: characterization of genetic variants in *SELPLG* and in nearby genes that are associated with PSGL-1 levels in a general population cohort; and to conduct Mendelian randomization studies that assess the directionality of causal relationships between PSGL-1 levels and proinflammatory cytokines or adhesion molecules.

## 2. Methods

### 2.1. Genome-Wide Association Study of Plasma PSGL-1

The largest and most recent GWAS with plasma measurements of PSGL-1 (*n* = 35,571 participants of European ancestry) was utilized, available from the UK Biobank Pharma Proteomics Project (PPP) [11]. Protein levels were quantified through antibody-based measures with the Olink (Uppsala, Sweden) Explore platform. Protein level values are unitless and represent protein abundance for an individual relative to the entire sample. Briefly, the UK Biobank—https://www.ukbiobank.ac.uk/ Last accessed 15 July 2026 PPP consortium sampled 54,219 participants, with ~85% randomly selected and the remaining specifically selected to enrich the sample for diseases and traits of interest. REGENIE v. 2.2.1 with imputed genotypes was used for GWAS analyses, accounting for population structure and standard covariates [12]. Summary statistics of the second largest publicly available GWAS with circulating levels of PSGL-1 (SCALLOP consortium) was utilized representing the genetic study of 90 cardiovascular proteins measured using the Olink (Uppsala, Sweden) PEA CVD-I panel [12]. This GWAS was a meta-analysis of GWAS from 13 cohorts, including up to 21,758 individuals of European ancestry.

### 2.2. Functional Mapping and Annotation (FUMA) of Genetic Associations

We used FUMA (version 1.4.1) [13], a functional mapping and annotation tool, to identify genomic risk loci. FUMA identifies independent variants reaching genome-wide significance (GWAS *p* < 5 × 10^−8^, r^2^ = 0.6) and selects lead variants independent from each other at r^2^ = 0.1 using 1000 Genomes phase III data for linkage disequilibrium (LD) calculations. By combining LD blocks 500 kb apart, genomic risk loci are defined, often identifying multiple and independent significant variants or lead variants at a single genomic locus.

### 2.3. In Silico Analysis—Genetic Function of SNPs Significantly Associated with Plasma PSGL-1

*SELPLG* was defined using the UCSC Genome Browser on Human (GRCh38/hg38) “https://genome.ucsc.edu/cgi-bin/hgGateway (accessed 13 July 2026)”. In silico analysis of the potential functionality of these alleles was conducted through dbSNP “https://www.ncbi.nlm.nih.gov/snp/ (accessed on 13 July 2026)” and Ensembl “https://may2025.archive.ensembl.org/index.html (accessed on 13 July 2026)”. We predicted the effects of promoter SNPs on transcription factor-binding sites and their impact on promoter activity using positional weight matrices search through Genomatix MatInspector 8.4 “https://www.eurenomics.eu/the-group/consortium/genomatix/index.html (accessed on 15 July 2024)” analysis and Jaspar “https://jaspar.elixir.no/ (accessed 13 July 2026)”.

### 2.4. Inflammatory Biomarkers

To assess other measures of inflammation, we used a GWAS of C-reactive protein (*n* = 204,402) [14], a GWAS meta-analysis of the inflammatory cytokines: SeSelectin, sICAM, and sVCAM (*n* = 13,365) [15] and GWAS summary statistics for glycoprotein acetyl levels (*n* = 115,078) [16]. Glycoprotein acetyl levels were obtained from the IEU GWAS as part of a metabolomic scan performed on a subset of UK Biobank participants. The GWAS meta-analysis of 47 cytokines, including SeSelectin, sICAM, and sVCAM, was based on three Finnish cohorts. The Northern Finland Birth Cohort 1966 [17] was quantified from fasting plasma samples using Bio-Rad’s BioPlex 200 system (Bio-Rad Laboratories, Hercules, CA, USA) with Milliplex Human Chemokine/Cytokine and CVD/Cytokine kits. The Cardiovascular Risk in Young Finns Study [18], quantified cytokines from serum samples using Biorad’s Bio-Plex Pro Human Cytokine 27-plex Assay and 21-plex Assay. The FINRISK study quantified cytokines as described for the Cardiovascular Risk in Young Finns Study [19].

### 2.5. Mendelian Randomization

Mendelian randomization (MR) is a method to assess the causal relationship between exposures and outcomes by leveraging the random allocation of alleles to offspring that occurs during gametogenesis. MR avoids residual confounding and reverse causation that may limit traditional observational studies. The assumptions of MR include that the genetic variants are robustly associated with the exposure, that they are not associated with other traits that may influence the outcome, and that they affect the outcome only through the exposure [20]. To ensure that our instruments were sufficiently powered, we calculated the F-statistic for each exposure SNP. Our primary MR estimate was the inverse-variance weighted (IVW) estimate since it is the most parsimonious and statistically powerful method. We also assessed results from the following sensitivity analyses: MR Egger, weighted median, simple median, and simple mode. We will consider MR findings as statistically significant if the IVW *p*-value < 0.001, based on a Bonferroni correction for a total of 49 inflammatory biomarkers tested, recognizing this is a conservative threshold given correlations among inflammatory biomarkers. We also consider the consistency of beta-coefficients across all MR sensitivity analyses.

## 3. Results

### 3.1. Multiple Cis and Trans Variants Significantly Associated with Plasma PSGL-1

**Figure 1:**
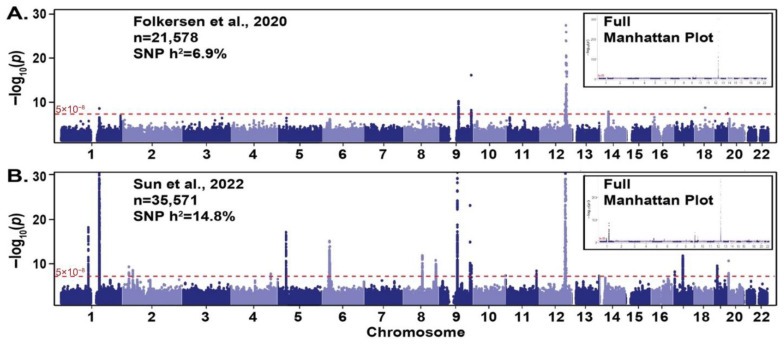
Genome-wide association studies in two general populations identifying loci associated with elevated plasma PSGL-1 levels. The inset shows the full Manhattan plot. We have zoomed in to better visualize the significant SNPs. (**A**). Zoomed and full GWAS by Folkersen et al. [12] and (**B**). Zoomed and full GWAS by Sun et al. [11].

Comparison of two GWAS studies of plasma PSGL-1 identified several loci associated with PSGL-1 in both GWAS (Figure 1). This includes a locus near the *TMEM119* and *SELPLG*s on chromosome 12, and two loci near the *GCNT1*, *ABO*, and *ST6GALNAC6* genes on chromosome 9 and a chromosome 1 locus near the *CD53* and *MTX1* genes. SNP-based heritability estimates are 6.9% and 14.8% for the Folkersen et al. [12] and Sun et al. [11] GWAS, respectively.

### 3.2. In Silico Analysis of Significant Coding and Promoter SELPLG SNPS Associated with Plasma PSGL-1 Levels

Three coding variants within the *SELPLG* coding region were identified, potentially leading to structural changes in the PSGL-1 protein. The genetic variants rs201689859 (GC>G), rs74792300 (T>C), and rs139943851 (G>C) produced a frameshift, synonymous, and missense change in the PSGL-1 protein, respectively and were associated with lower PSGL-1 plasma levels (beta values of −2.3882, −1.6689, and −1.7586, respectively).

Four variants were identified within the *SELPLG* promoter region and were associated with potential changes in promoter and transcription activity. The genetic variants rs1420663 (G>A), rs1833245 (T>A), rs1420664 (C>T), and rs8179110 (A>G) were located at 740, 678, 583, and 506 base pairs upstream of the transcription start site (TSS) of the *SELPLG*, respectively. In silico analysis revealed the rs1420664 (-583C>T) SNP to reside within a hypoxia-responsive element with the potential to alter binding of the hypoxia-inducible factor (HIF) transcription factor to the promoter. Additionally, rs8179110 (A>G) was situated at a proximal CpG promoter site potentially affecting the methylation/demethylation status and activity of the *SELPLG* promoter. These variants were associated with higher plasma PSGL1 levels (beta values of 0.2594, 0.1111, 0.0862, and 0.0849, respectively).

### 3.3. Top GWAS Genetic Variants Associated with Plasma PSGL-1 Levels

In silico analyses indicated the involvement of the following genes in an array of cellular signaling pathways: glycosylation [*ST6GALNAC6*]; membrane transport and trafficking [*VPS29*, *SVOP*]; immune and inflammatory responses [*CMKLR1*, *TMEM119*]; cell adhesion and cytoskeletal dynamics [*CMKLR1*, *CORO1C*, *SSH1*]; post-translational modifications, deubiquitination and protein stability [*PPP1CC*]; and metabolic and energy regulation [*ACACB*] (Table 1).

### 3.4. Functional Mapping and Annotation (FUMA) of Genetic Associations

**Figure 2:**
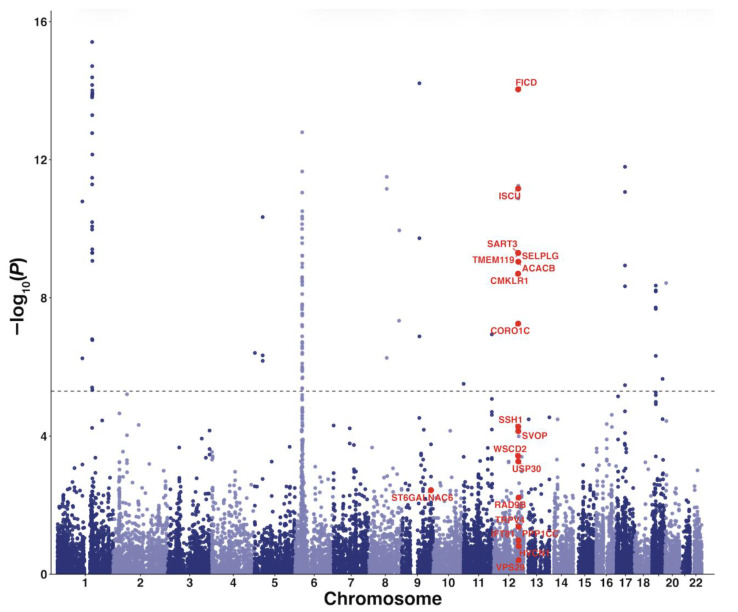
Independent variants reaching genome-wide significance (GWAS *p* < 5 × 10^−8^, r^2^ = 0.6) identified using FUMA GWAS. The location of SNPs/genes associated with plasma PSGL-1 levels are identified in red.

Functional mapping and annotation identified independent variants reaching genome-wide significance (GWAS *p* < 5 × 10^−8^, r^2^ = 0.6) in multiple genes associated with plasma PSGL-1 levels in the two GWAS studies [*FICD*, *ISCU*, *SART3*, *TMEM119*, *ACACB*, *CMKLR1*] (Figure 2).

### 3.5. Genetically Predicted Levels of Two Inflammatory Cytokines and Two Adhesion Proteins Are Associated with Plasma PSGL-1 Levels

**Figure 3:**
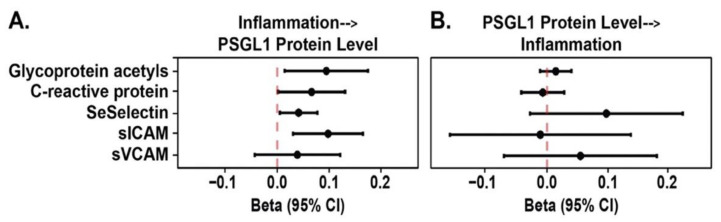
In bidirectional Mendelian randomization, genetically predicted levels of glycoprotein acetyls, CRP, SeSelectin, and sICAM, but not sVCAM, were associated with increased PSGL-1 levels (**A**). Genetically predicted plasma levels of PSGL-1 were not associated with elevated levels of these proteins (**B**).

We used summary statistics from a large-scale GWAS of 47 plasma proteins focusing on two proteins that are biomarkers of inflammation, C-reactive protein (CRP; *n* = 204,402) [14] and glycoprotein acetyls (GlycA; *n* = 115,078), and three adhesion proteins; soluble E-Selectin (SeSelectin), soluble vascular cell adhesion molecule-1 (sVCAM-1) and soluble intercellular adhesion molecule-1 (SICAM-1) [*n* = 13,365 participants] [15]. Bi-directional Mendelian randomization analyses of the association of the genetically predicted levels of inflammation markers with PSGL-1 levels found consistent evidence that genetically predicted levels of CRP, SeSelectin, GlycA, and SICAM-1 were associated with increased PSGL-1 levels (Figure 3). Sensitivity MR analyses revealed directions of effect consistent with the IVW estimate, and these were significant or nearly significant for SeSelectin, sICAM, glycoprotein acetyl, and CRP levels. This includes the MR-Egger estimates that are more robust to directional horizontal pleiotropy. In the reverse direction (with PSGL1 as exposure) there were no significant or consistent effects across all sensitivity analyses.

## 4. Discussion

In this large-scale genetic analysis leveraging two large and independent general population GWAS studies of plasma proteins, we identified both cis and trans genetic variants significantly associated with circulating levels of PSGL-1. Our results provide new insights into the genetic regulation of *SELPLG* expression and extend prior mechanistic and observational studies linking PSGL-1 to acute inflammation, vascular injury, and risk of ARDS [6,21,22,23]. Importantly, we demonstrate that both coding and promoter variants within *SELPLG* contribute to variability in plasma PSGL-1 levels, and in silico analyses suggest plausible mechanisms involving altered transcription factor binding and epigenetic regulation.

The finding that promoter variants such as rs1420664 (-583C>T) lie within hypoxia-responsive elements and may alter HIF binding supports prior observations that *SELPLG* is inducible by hypoxic and ROS-related pathways in preclinical ARDS models [8]. Similarly, rs8179110, located in proximity to CpG sites, may influence promoter methylation and gene activity, consistent with earlier reports of *SELPLG* regulation by epigenetic modifications [8]. These observations highlight potential molecular mechanisms through which environmental stressors, including hypoxia and oxidative stress, could interact with genetic variation to shape *SELPLG* expression and PSGL-1 levels.

Beyond cis-regulatory effects, we also identified multiple trans variants and pathways associated with PSGL-1, including genes involved in glycosylation, membrane trafficking, cytoskeletal remodeling, and immune signaling. For example, *ST6GALNAC6*, which encodes for alpha-N-acetylgalactosaminide alpha-2,6-sialyltransferase 6, belongs to a family of sialyltransferases that modify proteins and ceramides on the cell surface to alter cell–cell or cell-extracellular matrix interactions [24]. Given that PSGL-1 is a heavily glycosylated adhesion molecule whose function depends on post-translational modifications and interactions with selectins, these findings are biologically coherent and underscore the complexity of PSGL-1 regulation. Membrane transport and trafficking may impact PSGL-1 levels. Proteins such as vacuolar protein sorting (Vps29) encoded by the *VPS29* gene and SV2 related protein encoded by the *SVOP* gene regulate vesicular transport, potentially affecting the proper trafficking of PSGL-1 to the cell surface [25]. Any dysfunction in these trafficking pathways could impair PSGL-1 expression and its subsequent function in immune cell adhesion. Similarly, proteins such as SV2 related protein are involved in vesicular dynamics, which might impact how PSGL-1 is recycled within cells, further affecting its plasma levels [25,26]. In immune and inflammatory signaling, a G protein-coupled receptor protein encoded by the chemokine-like receptor 1 gene (*CMKLR1*) can modulate PSGL-1 activity to participate in immune cell recruitment, which could influence the levels of PSGL-1 during inflammation.

Regulation of the actin cytoskeleton by coronin actin-binding protein 1C, encoded by *CORO1C*, and slingshot protein phosphatase 1, encoded by *SSH1*, is potentially essential for PSGL-1’s role in cell adhesion, influencing PSGL-1’s ability to cluster on the cell surface, which is crucial for effective cell–cell interactions, particularly in the context of leukocyte adhesion during immune responses.

Post-translational modifications (PTMs), such as AMPylation by Fic Domain-Containing Protein Adenylyltransferase (*FICD* gene), can also impact PSGL-1 function and stability. PTMs are important for modulating protein stability, localization, and activity, thus affecting how PSGL-1 participates in immune responses. In addition, USP30, a deubiquitinating enzyme, can maintain PSGL-1 stability by preventing degradation, further emphasizing the importance of PTMs in regulating plasma PSGL-1 levels. Genes involved in metabolic regulation, such as *ACACB* encoding for acetyl-CoA carboxylase 2, could also indirectly affect PSGL-1 levels. Cellular metabolic states influence membrane lipid composition, which could affect membrane-bound proteins like PSGL-1.

Our Mendelian randomization analyses provide additional support for a causal link between systemic inflammation and elevated PSGL-1 levels. Specifically, genetically predicted levels of CRP, GlycA, soluble E-selectin, and sICAM-1 were associated with increased plasma PSGL-1 levels. These findings are consistent with clinical observations of PSGL-1 elevation in sepsis, ARDS, and COVID-19 and suggest that PSGL-1 may act as both a marker and mediator of systemic inflammatory signaling. Of note, we did not find evidence for reverse causation, i.e., genetically predicted PSGL-1 levels were not associated with downstream cytokine or adhesion molecule levels. Taken together, these results strengthen the premise that PSGL-1 functions as an integrative biomarker of inflammatory burden rather than an upstream driver of systemic inflammatory cytokine responses.

Our study has several strengths. We used two of the largest available GWAS of plasma proteins, providing robust statistical power to detect genetic associations. The use of complementary in silico functional annotation and MR analyses enabled us to move beyond association toward mechanistic insights and causal inference. Furthermore, the consistent identification of *SELPLG* variants across both GWAS reinforces the robustness of our findings.

Nevertheless, limitations should be acknowledged. First, plasma PSGL-1 levels were measured using antibody-based proteomic assays, which may not fully capture functional differences in PSGL-1 activity at the cellular level. Second, although MR reduces confounding, residual pleiotropy remains a possibility, particularly given the pleiotropic nature of immune pathways. Third, the GWAS populations were predominantly of European ancestry, which may limit the generalizability of our findings to more diverse populations. Fourth, although multiple MR sensitivity analyses were performed, residual pleiotropy remains possible. Finally, while our in silico analyses suggest functional consequences of specific SNPs, experimental validation is required to confirm their effects on transcription factor binding, promoter activity, and PSGL-1 expression.

## 5. Conclusions

Our findings demonstrate that both cis and trans genetic variation regulate circulating PSGL-1 levels and that inflammation-related biomarkers are causally linked to elevated PSGL-1. These results extend prior mechanistic and clinical observations, positioning PSGL-1 as a promising biomarker of inflammatory stress and a potential target for modulation in acute inflammatory syndromes, including ARDS and sepsis. Future work should focus on functional validation of identified variants, exploration of PSGL-1 as a therapeutic target, and assessment of its prognostic value in diverse patient populations.

## Figures and Tables

**Table 1:** Top genes carrying SNPs associated with PSGL-1 levels in both Folkerson et al. [12] [**A**] and Sun et al. [11] [**B**] GWAS studies.

(A) Genes with SNPs Associated with Plasma SELPLG (PSGL-1) (*p* < 10^−10^) (Folkerson et al. [12])							
Chr.				Name of Gene			
9				*ST6GALNAC6*			
12				*WSCD2*			
12				*CMKLR1*			
12				*FICD*			
12				*SART3* (Varview)			
12				*ISCU* (Varview)			
12				*TMEM119* (Varview)			
12				*SELPLG* (Varview)			
12				*CORO1C* (Varview)			
12				*SSH1* (Varview)			
12				*SVOP* (Varview)			
12				*USP30* (Varview)			
12				*ACACB* (Varview)			
12				*TRPV4* (Varview)			
12				*IFT81* (Varview)			
12				*VPS29* (Varview),			
12				*RAD9B* (Varview)			
12				*PPTC7* (Varview)			
12				*TCTN1* (Varview)			
12				*HVCN1* (Varview)			
12				*PPP1CC* (Varview)			
**(B) Genes with SNPs Associated with Plasma SELPLG (PSGL-1) (*p* < 10^−10^) (Sun et al. [11])**							
**Chr.**	**Name of Gene**	**Chr.**	**Name of Gene**	**Chr.**	**Name of Gene**	**Chr.**	**Name of Gene**
1	***CD53***	1	***YY1AP1***	6	***HCG27***	12	***SSH1***
1	***LRIF1***	1	***DAP3***	6	***HLA-C***	12	***DAO***
1	***DCST1***	1	***MSTO2P***	6	***HCG26***	12	***SVOP***
1	***ADAM15***	1	***GON4L***	6	***MICB***	12	***USP30***
1	***EFNA3***	1	***SYT11***	6	***LTA***	12	***UNG***
1	***SLC50A1***	1	***RIT1***	6	***EHMT2***	12	***ACACB***
1	***DPM3***	1	***KHDC4***	8	***ZC2HC1A***	12	***UBE3B***
1	***KRTCAP2***	1	***ARHGEF2***	8	***IL7***	12	***TRPV4***
1	***TRIM46***	1	***SSR2***	8	***ST3GAL1***	12	***IFT81***
1	***MUC1***	1	***UBQLN4***	9	***PCSK5***	12	***ATP2A2***
1	***THBS3***	1	***RAB25***	9	***RFK***	12	***FAM216A***
1	***MTX1***	1	***MEX3A***	9	***GCNT1***	12	***VPS29***
1	***GBAP1***	1	***LMNA***	9	***ST6GALNAC6***	12	***RAD9B***
1	***GBA***	5	***SPEF2***	9	***PIP5KL1***	12	***HVCN1***
1	***FAM189B***	5	***IL7R***	12	***WSCD2***	12	***PPP1CC***
1	***SCAMP3***	5	***CAPSL***	12	***CMKLR1***	12	***CUX2***
1	***CLK2***	5	***UGT3A1***	12	***FICD***	17	***GSDMA***
1	***HCN3***	6	***PSORS1C1***	12	***SART3***	17	***PSMD3***
1	***PKLR***	6	***CCHCR1***	12	***ISCU***	17	***CSF3***
1	***FDPS***	6	***TCF19***	12	***TMEM119***	17	***MED24***
1	***ASH1L***	6	***POU5F1***	12	***SELPLG***		
1	***MSTO1***	6	***PSORS1C3***	12	***CORO1C***		

## Data Availability

The data presented in this study are openly available in the GWAS catalog (64) and UKB-PPP at https://doi.org/10.7303/syn51364943. And here for inflammatory cytokines GWAS summary statistics: https://zenodo.org/records/7215468. Both of these accessed in 15 June 2023.
